# Pancreatic pseudocystwith stent placement in the background of narcotic use: a case report

**DOI:** 10.1186/1757-1626-1-219

**Published:** 2008-10-07

**Authors:** Stephen Offord, Bora Gumustop, Arthur Shepard

**Affiliations:** 1 Department of Family Practice, Saratoga Hospital, 211 Church Street, Saratoga Springs, NY 12866, USA; 2 Gastroenterologist, Albany Gastroenterolgy Consultants. PC, 1375 Washington Avenue, Suite 101, Albany, NY 12206, USA; 3 Medical Imaging, Information Systems Supervisor, Saratoga Hospital, 211 Church Street, Saratoga Springs, NY 12866, USA

## Abstract

A 49 year old gentleman presents with recurrent abdominal pain. The patient has a known history of chronic pancreatitis, alcoholism and narcotic addiction. Work-up, including computed tomography (CT) of the abdomen, demonstrated a 5.6 × 5.8 cm fluid collection contiguous with the pancreas. This was not seen on CT 18 months earlier. The patient's pain did not improve with bowel rest and pain control. He was transferred to another institution for endoscopic placement of a transgastric pancreatic stent. The procedure decreased the size the cyst and the patient's pain became more manageable.

## Introduction

Pancreatic pseudocysts have been recognized for over 185 years [1]. Today most are alcohol related and more than half are thought to resolve spontaneously [2]. This is a case of a 49 year old gentleman who develops a pancreatic pseudocyst that had to be surgically drained. Pancreatic pseudocysts have a unique physiology that does not lend itself easily to drainage [3]. Consequently management has tended towards watchful waiting provided the cyst is stable and asymptomatic [4]. Our patient also had narcotic dependence. Some evidence suggests at least a physiologic basis for the contribution of some narcotics towards the development of a pancreatic pseudocyst [5-7].

## Case presentation

### History of Present Illness

The patient was a 49 year old patient who presented with abdominal pain. He had visited our emergency room 13 times in the last year and this would be his fourth admission. His abdominal pain was constant, mid-epigastric, and exacerbated with eating. Why the pain flared up this time was unclear. He did not consume any alcohol recently or change his diet. He denied chest pain, shortness of breath, melena and fever. He had not vomited but was nauseated.

### Past Medical History

His only medical problem was chronic pancreatitis and its complications. His pancreatitis was initially precipitated by heavy alcohol use. In recent years he no longer drank alcohol but was addicted to lortab. We were informed that at one time he would take up to 30 or 40 pills a day of lortab purchased illegally off the street.

### Past Surgical History

None

### Past Social History

The patient smoked one pack per day. He no longer drank alcohol. He was disability for chronic pancreatitis. He denied drug use at this time, including non prescribed narcotics.

### Past Family History

His mother passed away from coronary artery disease. As far as he knew, it did not develop when she was young.

### Medications

His narcotic use varies. Presently he was taking lortab 5/500 mg, four tablets a day. He also took Viokase-8 tablets, four before meals and two before snacks.

### Physical Exam

Vital signs: Afebrile. Pulse 77. Respiratory rate 18. Blood pressure 135/73. Oxygen saturation 100% on room air.

In general he seemed uncomfortable. He sat still and winced when he talked. Head exam: Normal cephalic, atraumatic. Ears and throat were normal. He had poor dentition although he still has his own teeth. Neck: I did not note lymphadenopathy or a carotid murmur. Heart: regular rate and rhythm. No murmur was noted. Lung exam: clear with good air movement. Abdomen: Tenderness with palpation in the epigastric region. There was no guarding or rebound tenderness. His abdomen was not rigid. Bowel sounds were quiet. Extremities: no edema and no clubbing of nails.

### Laboratory

His amylase was elevated at 239 IU/L. White cell count was 10.4 × 10^3^/*u*L with 76% neutrophils. Hemoglobin was 14.6 g/dL. His metabolic panel was normal. Calcium was 8.7 mg/dL and his an albumin was 3.4 g/dL. His triglycerides were 128 mg/dL. His alcohol level was 0.

**Figure 1:**
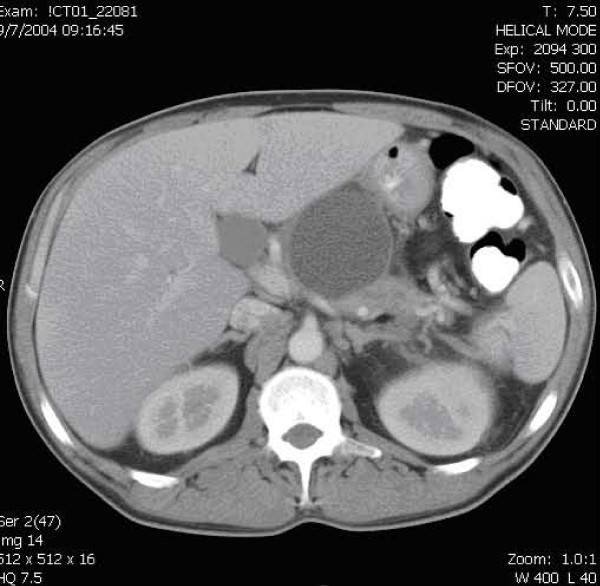
CT of the abdomen showing a large 5.6 × 5.8 cm fluid collection contiguous with the body of the pancreas.

Computed tomography (CT) with contrast of the abdomen and pelvis (figure 1) showed a large 5.6 × 5.8 cm low attenuation collection with a well-defined wall contiguous with the body of the pancreas. Mild fat stranding was also noted around the pancreatic tail.

**Figure 2:**
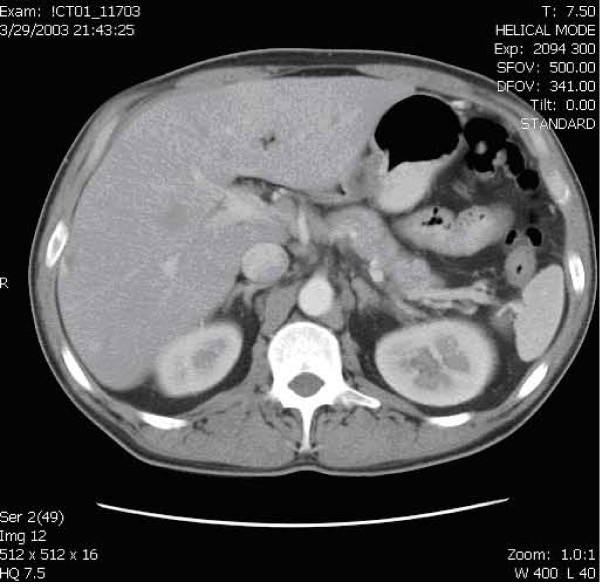
CT of the abdomen of the same patient 18 months earlier. No pancreatic fluid collection is seen.

A CT of the abdomen 18 months earlier (figure 2) demonstrated no pseudocyst or pancreatic inflammation.

### Hospital course

The patient was admitted for bowel rest and pain control. Because of continued abdominal pain, several days passed and he still was unable to tolerate oral intake. Total parenteral nutrition was provided. Follow up CT scans of the abdomen on hospital days 7 and 14 showed no change.

Finally, on hospital day 24 he was transferred to another institution for pseudocyst drainage and stent placement. He underwent transgastric endoscopic ultrasound and fluoroscopy guided pseudocyst drainage with stent placement.

### Outcome

**Figure 3:**
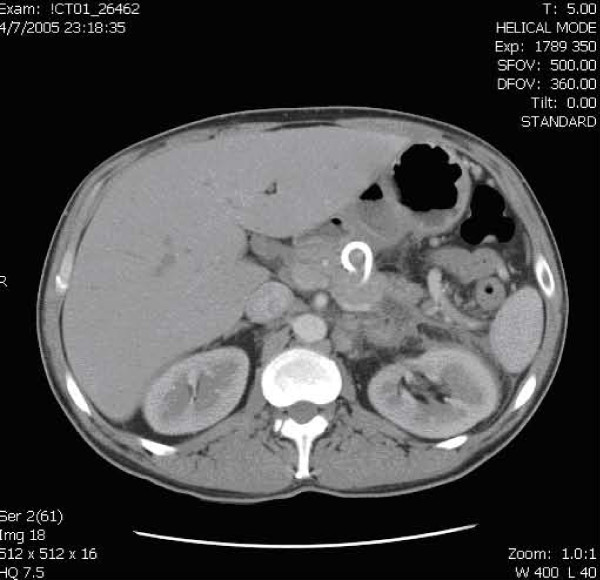
CT of the abdomen 7 months later after insertion of the transgasticstent. The pseudocyst now measures just 17 mm in size and the stent is still in place.

The patient's pain improved following stent placement. Following discharge, the patient continued to have periodic admissions for flare ups of abdominal pain, but they became less frequent. Figure 3 shows a CT of the patient's abdomen seven months later. The pseudocyst measures 17 mm in size with the stent still in place. The patient was also enrolled in a methadone program to address his narcotic addiction.

About two years after this admission the patient expired. He died at his home suddenly. Autopsy reports concluded cause of death to be a drug overdose, methadone and hydrocodone. It also noted severe atherosclerosis of the coronary arteries.

## Discussion

Pancreatic pseudocysts develop after acute pancreatitis 5 to 15% of the time, and more than half are thought to resolve spontaneously. Most (~65%) are alcohol related [2]. While true cysts have an epithelial lining that secretes the cystic fluid, pancreatic pseudocysts are filled with exocrine secretions from a disruption of its own ductal system. The body reacts to the hostile, digestive enzymes-amylase, lipase and others, with inflammation. A fibrous wall forms over several weeks to contain the spill and protect the surrounding tissue [2,3].

Persistence of the cyst implies an ongoing communication with the disrupted pancreatic duct [3]. The patient's chronic abdominal pain was likely due, at least in part, to the development of a pseudocyst. Pseudocysts seem more likely to persist in patients who continue to drink alcohol [2,4]. Their persistence in narcotic users is less appreciated.

Pancreatic pseudocyst management has tended towards watchful waiting [3,8,9]. The first planned operation of a pancreatic cyst was in 1822 in Prague by Carl Gussenbauer [1]. He palpated an 8.5 × 22 cm swelling in the epigastrium of a 40 year old man who recently drank 7 liters of beer. An abdominal incision was used to drain 1900 cc of grayish, black fluid. The patient improved but later developed an external pancreatic fistula. He was discharged against medical advice on post-operative day 84.

Back then, pancreatic pseudocysts were diagnosed by physical exam [1]. Consequently, cysts had to be quite large before they could be found. Even with the help of an upper GI series, introduced in 1910 [10], any cysts discovered were big, and spontaneous resolution was thought to be rare (less than 10%). As a result, most that were discovered were drained [1].

Two conservative trends followed and each was driven, in part, by advances in imaging [3]. The first trend occurred one hundred and fifty years after Gussenbauer's partial success. In 1979, Bradley and colleagues followed the natural history of pseudocysts by ultrasonography. They noted cysts less then 6 cm had a 40% spontaneous resolution rate and a very low complication rate (20%). Pseudocysts present more than 6 weeks, however, almost never resolved and had a very high complication rate (46%). Consequently there was a strong trend to observe smaller, younger pseudocysts [8]. Surgical drainage was reserved for cysts 'greater than 6 cm or present for more than 6 weeks.'

The second and latest trend followed a study using computed tomography (CT) imaging [9]. In the late 1980's, Yeo et al observed the natural history of pseudocysts by CT. The '6 cm or 6 week' paradigm began to falter. He noted for, example, 36% of pseudocysts greater than 8 cm could be managed without surgery. Later, Vitas and Sarr described 68 patients with pancreatic pseudocysts managed expectantly for as long as 116 months (mean 51 months). Serious complications were seen in 9% of this expectant group compared to 10% of patients who had elective operations in the primary treatment group [4]. Now there is no longer a cut and dried quantitative policy for surgery. Patients with pseudocysts of any size or duration can be managed without drainage provided they are symptom free, and provided the cyst size is stable or declining [3,11]. Our patient was not symptom free, and because of his pain, we were unable to observe him for more than a few weeks.

Most pancreatic pseudocysts are drained internally with an open surgical approach [11]. Open surgery is indicated for recurrent pseudocysts, cysts complicated by ductalstenosis, and in suspected neoplasm with need for biopsy. Endoscopic approaches are becoming increasingly popular but require the cyst be firmly adherent to the gastrointestinal tract and bulging into the lumen. For mature cysts, in experienced hands, endoscopy should be given first choice. Percutaneous drainage is reserved primarily for infected cysts and has a 10% risk of a persistent pancreatic fistula. ERCP is often done first to define pancreatic duct anatomy and confirm ductal communication with the cyst [2]. Our patient, as noted, had an endoscopic approach.

Our patient's care was complicated by narcotic use. I could find no evidence in the literature for an increased risk of pseudocyst with prolonged narcotic use. It is at least theoretically possible. Morphine is known to increase pancreatic ductal pressure at the sphincter of Oddi [5,6]. And there are conflicting reports about morphine's effects on pancreatic secretion. Dooley et al demonstrated morphine inhibited pancreatic secretion into the duodenum [12]. But in an earlier study, Gullo et al showed morphine actually increased volume of water and electrolyte secretion from the pancreas [7]. The discrepancy could be explained by their respective methods. Dooley at al measured duodenal aspirates while Gullo et al inserted a polyethylene tube directly into Wirsung's canal. It is possible that by simultaneously increasing sphincter of Oddi pressure, morphine decreases pancreatic secretion if measured distally to the sphincter (in the duodenum) but in fact increases intrapancreatic secretion over all. Increasing both ductal pressure and intra-pancreatic secretion could favor development of a pseudocyst. Interesting, tramadol, which was shown to decrease sphincter of Oddi pressure, has been rated as a better analgesic for chronic pancreatits pain compared to morphine [13].

## Conclusion

Pancreatic pseudocysts should be considered in the differential of any patient with chronic abdominal pain, especially if he or she has a history of chronic pancreatitis. Their unique physiology, evolution of management, and potential relation to narcotic use make them worthy of review. Further clarifying any potential role of narcotics in pancreatic pseudocyst development would be important. Many patients manage the pain of chronic pancreatitis with narcotics.

## Competing interests

The authors declare that they have no competing interests.

## Authors' contributions

SO drafted the manuscript. BGprovided insight into the discussion. AS provided technical assistance with the CT images. All authors read and approved the manuscript.

## Consent

Written informed consent was obtained from the patient's Health Care Agent for publication of this case report and accompanying images. A copy of the written consent is available for review by the Editor-in Chief of this journal.
